# Reversal agents for oral anticoagulant-associated major or life-threatening bleeding

**DOI:** 10.1007/s11739-019-02177-2

**Published:** 2019-08-24

**Authors:** Marco Moia, Alessandro Squizzato

**Affiliations:** 1 Fondazione IRCCS Ca’ Granda Ospedale Maggiore Policlinico, Angelo Bianchi Bonomi Hemophilia and Thrombosis Center and Fondazione Luigi Villa, Milan, Italy; 2 Research Center on Thromboembolic Disorders and Antithrombotic Therapies, University of Insubria, Varese, Italy; 3 U.O.C. Medicina Generale, Ospedale Sant’Anna - ASST Lariana, via Ravona 20, 22042 San Fermo della Battaglia, Como Italy; 4 Department of Medicine and Surgery, University of Insubria, Como, Italy

**Keywords:** Bleeding, Idarucizumab, Andexanet, Prothrombin concentrate complex, Direct oral anticoagulant, Vitamin K antagonist

## Abstract

Oral anticoagulants (OA) are effective drugs for treating and preventing the formation of blood clots in patients with atrial fibrillation, mechanical heart valves and venous thromboembolism but their therapeutic effect is always counterbalanced by an increased risk of bleeding. Direct oral anticoagulants (DOACs) have brought advantages in the management of many patients, with evidence of a lower risk of intracranial bleeding in comparison to vitamin K antagonists (VKAs). However, due to the increased number of anticoagulated patients worldwide, major and life threatening OA-related bleeding is also increasing, and effective reversal strategies are needed. We reviewed the reversal strategies for both VKAs and DOACs in the light of the latest evidence and recent guidelines, taking into account non-specific methods with fresh frozen plasma (FFP), prothrombin complex concentrate (PCC) or four factor PCC, as well as specific reversal antidotes that are already approved or in approval phase. Most published studies on OA reversal have drawbacks, such as lacking a control arm or data on clinically relevant outcomes, and current guidelines’ recommendations are mainly based on panellists’ judgment. There is an urgent need for well-designed studies in this field. In the meanwhile, to improve the correct use of available resources and patients’ outcomes, we suggest a seven-element bundle for an optimal management of OA-associated major bleeding, including the implementation of fast turnaround time for laboratory tests in emergency, i.e. INR and DOAC plasma levels, and to build up a ‘bleeding team’ that includes experts of hemostasis, lab, trauma, emergency medicine, endoscopy, radiology, and surgery in every hospital.

## Introduction

The beneficial effect of anticoagulant drugs, vitamin K antagonists (VKAs) and direct oral anticoagulant (DOACs) is always counterbalanced by an increased risk of bleeding. As almost 2% of the general population of United States and Western Europe are receiving an anticoagulant drug, bleeding during anticoagulation is a common event. In clinical practice, the annual rate of VKA-associated and DOAC-associated major bleeding is reported approximately between 1.5 and 3.5%, and much higher for minor bleeding [1–4]. There are differences on the rate of bleeding due to indications (atrial fibrillation, venous thromboembolism, and heart valves prosthesis), patients’ characteristics (age, sex, co-morbidities and co-medications), quality of treatment (e.g. time spent in therapeutic range [TTR]) and adherence [2–4].

## A seven-element bundle for oral anticoagulant-associated major or life-threatening bleeding

It is counteractive that reversal agents may be beneficial only when the administration is integrated in a multimodal approach to the bleeding patient on oral anticoagulant (OA). Based on our experience and published guidelines (see Tables 1, 2), we propose a seven-element bundle for treating OA-associated major or life-threatening bleeding:

1. withdrawal of the anticoagulant drug till local hemostasis is safe;
2. fluid replacement, to support the cardiovascular system and renal function;
3. blood tests, to check for hemoglobin level, platelet count, renal function, liver function, PT, aPTT (and DOAC plasma level, when necessary);
4. red blood cell transfusion, platelet and/or fresh frozen plasma transfusion, and eventually tranexamic acid;
5. any local hemostatic measure, such as endoscopy, interventional radiology procedure or surgical intervention;
6. check and management of any additional bleeding risk factor, such as uncontrolled hypertension, excessive alcohol intake, acute renal insufficiency, low platelet count, antithrombotic therapies, in particular antiplatelet drugs, non-steroidal anti-inflammatory drugs (NSAIDs) and glucocorticoids;
7. reversal of the anticoagulant effect.

**Table 1:** Reversal agents in case of life-threatening bleeding (adapted from Steffel et al. [2])

Direct thrombin inhibitors (dabigatran)	FXa inhibitors (apixaban, edoxaban and rivaroxaban)
Specific reversal Idarucizumab 5 g i.v. in two bolus doses of 2.5 g i.v. no more than 15 min apart	Specific reversal Andexanet alpha (if available and approved) Bolus over 15–30 min, followed by 2-h infusion Rivaroxaban (last intake > 7 h before) or apixaban: 400 mg bolus, 480 mg infusion @ 4 mg/min Rivaroxaban (last intake < 7 h before or unknown) or enoxaparin or edoxaban: 800 mg bolus, 960 mg infusion at 8 mg/min
Non-specific reversal treatment (if/when specific reversal are not available) Prothrombin complex concentrate (PCC) 50 U/kg (with additional 25 U/kg if clinically needed) 4 factor prothrombin complex concentrate (PCC) 50 U/kg Activated PCC (aPCC) 50 U/kg; max 200 U/kg/day): no strong data about additional benefit over PCC. Can be considered before PCC, if available

**Table 2:** 7-Element bundle for managing OA-associated major or life-threatening bleeding

1	Cessation of the OA
2	Fluid replacement, to support the cardiovascular system and renal function
3	blood tests, to check for hemoglobin level, platelet count, renal function, liver function, PT, aPTT, and DOACs’ plasma level
4	Red blood cell transfusion, and eventually platelet and/or fresh frozen plasma transfusion when necessary; consider tranexamic acid
5	Any local hemostatic measure, such as endoscopy, interventional radiology procedure or surgical intervention
6	Check and management of any additional bleeding risk factor, such as uncontrolled hypertension, excessive alcohol intake, acute renal insufficiency, low platelet count, antithrombotic therapies, in particular antiplatelet drugs, NSAIDs and glucocorticoids
7	If DOAC: plasma measurement of the DOAC level and reversal agent administration (idarucizumab, Andexanet alfa or 4F-PCC) only when the anticoagulant drug is active in patient's plasma in measurable quantities If VKA: INR measurement and vitamin K administration plus reversal with PCC (FFP if PCC unavailable)

*OC* oral anticoagulant, *DOAC* direct oral anticoagulant, *VKA* vitamin K antagonist, *4F-PCC* 4-factor prothrombin complex concentrate, *INR* international normalized ratio, *FFP* fresh frozen plasma

We summarized the available evidence on this seventh element in the following paragraphs. Reversal agents for VKAs are recommended in case of major bleeding, in particular for life-threatening events, and according to INR level. Specific reversal agents for DOACs are indeed recommended in case of life-threatening bleeding and when the anticoagulant drug is active in patient's plasma in measurable quantities.

## Reversal of VKA-associated major or life-threatening bleeding: vitamin K, fresh frozen plasma, and prothrombin complex concentrate

Reversal of VKA is recommended in major bleeding events and, in case of life-threatening events, such intracranial hemorrhage, it should be done as soon as possible [5]. Table 3 reports the International Society of Thrombosis and Haemostasis (ISTH) definition of major bleeding [6]. This strategy should also be applied to patients with indication to some urgent surgical or other high bleeding risk procedure, like spinal puncture, since intravenous vitamin K administration takes several hours to normalize INR values, an additional reversal agent has to be infused. In these clinical settings, recommendations for prompt reversal are reported in most guidelines [2–4].

**Table 3:** ISTH definition of major bleeding [6]

1. Fatal bleeding, and/or
2. Symptomatic bleeding in a critical area or organ, such as intracranial, intraspinal, intraocular, retroperitoneal, intra-articular or pericardial, or intramuscular with compartment syndrome, and/or
3. Bleeding causing a fall in hemoglobin level of 20 g L^−1^ (1.24 mmol L^−1^) or more, or leading to transfusion of two or more units of whole blood or red cells

Rapid reversal can be achieved by the administration of fresh frozen plasma (FFP) or non-activated prothrombin complex concentrates (PCCs) in addition to administration of intravenous vitamin K [2–5]. Vitamin K (at least, 10 mg intravenously, slow infusion) should always be administered to prevent loss of effectiveness of reversal over time, due to the half-life of the transfused coagulation factors.

Two types of PCCs are commonly available: they are lyophilized concentrates, containing three vitamin K-dependent coagulation factors (referred as 3-F, containing factor II, IX and X, and only small amounts of factor VII), or four vitamin K-dependent factors (referred as 4-F, containing factor II, VII, IX and X). PCCs have several advantages over plasma in reversal of VKA, since they can be administered promptly, in small volumes, without need of thawing or blood type matching.

However, the evidence on the efficacy and safety of reversal procedures is still based on clinical experience more than on sound evidence of net clinical benefit, because RCTs are available for some subset of anticoagulated patients. Three randomized controlled trials comparing PCCs vs FFP were published in patients with life-threatening bleeding during VKA treatment [5, 7, 8]. Overall, patients receiving 4-F PCCs achieved a more rapid INR normalization but a possible increased risk of any thromboembolism in comparison to FFP. In addition, there was no difference in mortality between those receiving 4-F PCCs and FFP (relative risk [RR], 0.92, 95% confidence interval [CI] 0.37–2.28; absolute risk reduction [ARR], 10 fewer deaths per 1000, 95% CI 78 fewer to 159 more per 1000) [4]. Four-factor PCC reduced the incidence of volume overload (RR, 0.34, 95% CI 0.13–0.85; ARR, 107 fewer episodes per 1000, 95% CI 24 fewer to 141 fewer per 1000) [4]. Benefits and harms with 4-F PCC vs FFP were balanced based on very low certainty evidence: the relatively small number of included patients and events does not allow definitive conclusion [5, 7, 8]. Reduction of potentially severe transfusion reactions and/or circulatory overload may counterbalance the higher cost of PCCs compared to FFP; in addition, FFP requires additional staff time to be prepared and administered.

Guidelines suggest that patients with major or life-threatening VKA-associated bleeding should be promptly treated with 4-F PCCs at doses tailored on INR value in addition to intravenous vitamin K (see Table 4) [5]. FFP should be used as first line agent in case PCCs are not immediately available.

**Table 4:** Prothrombin complex concentrate or fresh frozen plasma doses in patients with major bleeding used in the study by Sarode and colleagues [5]

Baseline INR	4F-PCC dose, IU per kg body weight^a^	Fresh frozen plasma mL per kg body weight^a^
2 to ≤ 4	25	10
4 to ≤ 6	35	12
> 6	50	15

Maximum dose ≤ 5000 IU of 4F-PCC or ≤ 1500 mL FFP

*4F-PCC* 4-factor prothrombin complex concentrate, *INR* international normalized ratio

^a^Dose calculation based on 100 kg body weight for patients weighing > 100 kg.

INR should always be checked at the end of PCC (or FFP) infusion and after 8–12 h, to assess the efficacy of drug reversal: supplementation with PCC or FFP should be considered if INR is not completely normalized. Unfortunately, no studies compared less expensive 3-F PCCs to 4-F PCCs; in our experience, 3-F PCCs can correct INR in patients anticoagulated with VKA and can be used instead of FFP, when 4-F PCCs are not available.

## Reversal of DOAC-associated major or life-threatening bleeding

### Idarucizumab

In 2015 idarucizumab was approved as specific reversal agent for dabigatran and it has become the standard of care for the reversal of dabigatran [2–4]. Idarucizumab is a humanized monoclonal antibody fragment, it binds irreversibly to free and thrombin-bound dabigatran within few minutes. It is administered as two consecutive rapid bolus doses of 2.5 g intravenous, no more than 15 min apart [3]. The efficacy and safety of idarucizumab was mainly shown in the RE-VERSE AD (Reversal Effects of Idarucizumab on Active Dabigatran) study, a phase 3, prospective, cohort study in dabigatran-treated patients who present with uncontrolled or life-threatening bleeding (group A) or non-bleeding patients who require emergent surgery or invasive procedure (group B). No drug activity testing was required before administering idarucizumab to avoid delays in treating patients with uncontrolled or life-threatening bleeding. Most patients (95.7%) from 39 countries were treated with dabigatran to prevent AF-associated stroke. The median age of participants was 78 years. The median maximum percent of blood test reversal was 100% within 4 h following idarucizumab administration on the basis of either the diluted thrombin time or the ecarin clotting time; the median time to cessation of bleeding was 2.5 h and the median time to initiation of procedure in patients requiring surgery was 1.6 h. At 90 days, thrombotic events had occurred in 6.3% of the patients in group A and in 7.4% in group B, and the mortality rate was 18.8% and 18.9%, respectively (see Table 5). Almost two-thirds of patients had not had their anticoagulants restarted at the time of their thrombotic event.

**Table 5:** Comparison of major oral anticoagulation-reversal studies

Trial	Reversal agent	Anticoagulant	Number of patients		Hemostatic Efficacy (95% CI)	Thrombotic event rate at 30 days (%)	Mortality at 30 days (%)
			Total	% ICH			
ANNEXA-4 (2019)	Andexanet	FXa inhibitors (enoxaparin, apixaban, edoxaban, rivaroxaban)	352	64	82% (77–87)	10	14
RE-VERSE AD (2017)	Idarucizumab	Dabigatran	301	32.6	67.7%^a^	4.8	12
Sarode (2013)	4F-PCC	Warfarin	98	12	72% (64–81)	7.8^b^	5.8
	Plasma		104	12	65% (56–75)	6.4^b^	4.6

*4F-PCC* four factor prothrombin complex concentrate

^a^Hemostatic efficacy reported only in patients without intracranial hemorrhage

^b^Events evaluated at 45 days

Notably, nine patients received an additional dose of idarucizumab due to recurrent bleeding or the need for a second urgent procedure, which was related to recurrent prolongation of clotting time due to redistribution of unbound dabigatran from the extravascular to the intravascular compartment [9, 10].

## Andexanet alpha

Andexanet alpha, also called factor Xa (recombinant), inactivated-zhzo, was recently approved in the United States and in Europe [3]. Andexanet alpha is a modified human recombinant FXa decoy protein that lacks catalytical activity. It is a specific reversal agent that binds with high affinity not only to direct Xa inhibitors, but also to the indirect FXa inhibitors, like low-molecular-weight heparin and fondaparinux [3].

Results of two randomised, double-blind, placebo-controlled, phase II trials (ANNEXA [Andexanet Alfa, a Novel Antidote to the Anticoagulation Effects of FXA Inhibitors trials]) performed in healthy volunteers aged 50 to 75 years who received apixaban (ANNEXA-A) and rivaroxaban (ANNEXA-R) were published [11]. A higher dose of andexanet was used for rivaroxaban than for apixaban because of higher plasma concentrations and a larger volume of distribution.

Anti-FXa activity was rapidly (within 2–5 min) reduced by 92% to 94% with andexanet bolus with the reduction of 20% observed in the placebo group. Following a dose of andexanet there is a fall of anti-Xa activity followed by a slow return of the anticoagulant effect of FXa inhibitor over time. Of note, andexanet alpha had a good safety profile, including no evidence for the generation of neutralizing antibodies [11].

The ANNEXA-4 phase 3b to 4 study assessed the efficacy and safety of andexanet alpha in patients treated with FXa inhibitors with acute major bleeding, but not in those anticoagulated subjects who require emergency or urgent procedures [12, 13]. Because of its pharmacodynamic half-life of 1 h, the drug was administered as a bolus over 15–30 min, followed by a 2-h infusion. The dosing depends on the DOAC and on the timing since last intake: for rivaroxaban (with the last intake > 7 h before reversal) or apixaban, a 400 mg bolus is administered followed by a 480 mg infusion (4 mg/min). For rivaroxaban (with the last intake < 7 h before reversal or unknown recent intake), edoxaban or enoxaparin, an 800 mg bolus followed by a 960 mg infusion (8 mg/min) is given. A descriptive analysis of 67 patients was preliminary published: 12 h after the andexanet infusion, clinical haemostasis was adjudicated as excellent or good in 37 of 47 patients in the efficacy analysis (79%; 95% CI 64–89). Thrombotic events occurred in 12 of 67 patients (18%) during the 30-day follow-up. In the final paper, 352 patients who had acute major bleeding within 18 h after administration of a FXa inhibitor were described. Twelve hours after the andexanet infusion, clinical haemostasis was adjudicated as excellent or good in 204 of 249 patients (82%; 95% CI 77–87). Thrombotic events occurred in 34 patients (10%) during the 30-day follow-up (see Table 5) [13].

## Non-specific reversal agent: PCC and activated PCC

Animal experiments as well as studies in healthy volunteers have indicated the potential usefulness of PCCs and activated PCCs (aPCC) for DOAC reversal [2]. However, the efficacy on clinical outcomes of PCC or aPCC in DOAC-associated bleeding has not been investigated in a RCT. In particular, to the best of our knowledge, no phase III RCT comparing PCCs to specific drugs, i.e. idarucizumab or andexanet, has been performed or is currently ongoing/planned.

Data from the large phase III RCTs demonstrated that outcomes of bleedings under DOACs were similar than in the VKA arm with similar treatment used (including PCC/aPCC) [2]. In addition, several observational studies in patients mayor DOAC-associated bleeding have been published, suggesting that (a)PCCs seems to be efficacious in supporting haemostasis [2, 14, 15]. Among them, edoxaban effects on bleeding following punch biopsy and reversal by a 4F-PCC was investigated in 110 subjects, suggesting that a dose of 50 IU/kg 4F-PCC may be suitable for reversing edoxaban anticoagulation [16].

## Guidelines

The most recent European Heart Rhythm Association (EHRA) guidelines for patients with atrial fibrillation recommend to use idarucizumab in case of life-threatening bleeding, and to consider it for non-life-threatening major bleeding. They recommend a continued clinical and laboratory monitoring, since a 5 g dose of idarucizumab may not completely neutralize a very high level of dabigatran (e.g. in case of overdose or renal insufficiency). Moreover, low levels of dabigatran may reappear after 12–24 h. In addition, when immediate procedures (such as immediate life-, limb- or organ-saving intervention, typically cardiac, vascular, and neurosurgical emergency procedures) need to be performed within minutes of the decision to operate and cannot be delayed, EHRA guidelines suggest that reversal with idarucizumab should be considered, especially in moderate- to high-haemorrhagic risk procedures [2]. Consistently with EHRA, the recently published ASH guidelines for patients with venous thromboembolism (VTE) suggest using idarucizumab in addition to cessation of dabigatran rather than no idarucizumab for patients with life-threatening bleeding during dabigatran for treatment of VTE; some panel members were concerned about the possibility of VTE following idarucizumab administration [4].

The last edition of the EHRA guidelines hypothesizes that andexanet alpha may become the first choice of therapy in life-threatening bleeding under FXa-inhibitor therapy in patients with atrial fibrillation (pending its regulatory approval and availability). In addition, the panelists suggest that the administration of PCC or aPCC can be considered in a patient with life-threatening bleeding under FXa-inhibitor therapy in patients with atrial fibrillation if immediate haemostatic support is required, especially in situations where a specific reversal agent is not available. The choice between PCC and aPCC may depend on their availability and the experience of the treatment centre. Particularly aPCC induces a strong pro-coagulant effect; therefore, they should only be administered by physicians experienced in their use and in bleeding that cannot be stopped in any other way [2].

The recently published ASH guidelines suggest using either andexanet alpha in addition to cessation of oral direct Xa inhibitor rather than no andexanet alpha, or 4-factor PCCs administration as an addition to cessation of oral direct Xa inhibitor, or cessation of oral direct Xa inhibitor alone in patients with life-threatening bleeding during oral direct Xa inhibitor treatment of VTE. These recommendations do not apply to non-life-threatening bleeding because the panelists consider that cost likely outweighs potential benefit and there is also a small but quantifiable increased risk of thromboembolism associated with administration of PCCs. The guideline panel offers no recommendation for one approach over the others because of the lack of direct comparison efficacy studies between the two approaches [4].

## Plasma level of DOACs: useful or necessary?

The administration of a reversal agent can be useful only when the anticoagulant drug is active in patient's plasma in measurable quantities. This means that the measurement of plasma levels of DOACs can be necessary in the management of DOAC-associated bleeding such as INR for VKA-associated bleeding. Normal results of dTT/ecarin clotting time (for dabigatran) and anti-Xa activity (for anti-FXa treated patients) likely exclude relevant activity of these anticoagulant drugs in patients' plasma. Due to the different pharmacokinetic properties of DOACs and of antidotes, and to the different half-life of blood coagulation factors (produced by the patient or administered by PCC or FFP), INR for VKAs and plasma levels for DOACs should be measured after the administration of reversal agents to carefully check whether reversal strategies have been consistently effective. As a matter of fact, reappearance of anticoagulant activity of anti-Xa drugs may occur after stopping the infusion of andexanet-alpha and, less frequently, after reversal of dabigatran with idarucizumab. Finally, knowledge of DOAC plasma level during major or life-threatening bleeding may be relevant for the subsequent decision to re-start or not OA. If and when resume OA is indeed a multi-parametric choice, and knowledge of an over-therapeutic or sub-therapeutic plasma levels of DOAC—such as INR level for VKA—is certainly relevant for the decision to re-start or not OA.

## Building up a ‘*bleeding team’*

Guidelines to treat major or life-threatening OA-related bleeding should be known and applied in every hospital, mainly in the emergency departments. However, some reports show a disappointing application of the existing guidelines in clinical practice [17]. The outcome of patients with OA-related bleeding can be improved not only by the use of reversal agents, but also by surgical, endoscopic, endovascular procedures and by an intensive general support applied in a timely and integrated strategy. Our clinical experience supports the building up of a ‘bleeding team’ that includes experts of hemostasis, lab, trauma, emergency medicine, endoscopy, radiology and surgery. This team should take the responsibility of implementing international guidelines based on local organization and resources, to update hospital procedures, to analyze and audit the results.

## Conclusion

Urgent complete reversal of the anticoagulant effect is necessary in case of major or life-threatening bleeding, trauma, emergency surgery or invasive procedures. Effective strategies to reverse the anticoagulant effects of VKAs and DOACs are now available. However, many of the published studies on anticoagulation reversal have several methodological drawbacks, such as lacking data on clinically relevant outcomes. Therefore, as no solid evidence is available, many current guidelines’ recommendations are mainly based on panellists’ judgment [2, 4].

We proposed a seven-element bundle for treating OA-associated major or life-threating bleeding: haemostasis experts, lab experts, emergency physicians/intensivists, endoscopy experts, radiologists and surgeons of different specializations should work together in multidisciplinary ‘bleeding team’. It is advisable that every hospital implements such a ‘bleeding team’ to optimize management of bleeding patients.
