# Correction to: Reversal agents for oral anticoagulant-associated major or life-threatening bleeding

**DOI:** 10.1007/s11739-019-02232-y

**Published:** 2019-11-27

**Authors:** Marco Moia, Alessandro Squizzato

**Affiliations:** 1 Fondazione IRCCS Ca’ Granda Ospedale Maggiore Policlinico, Angelo Bianchi Bonomi Hemophilia and Thrombosis Center and Fondazione Luigi Villa, Milan, Italy; 2 Research Center on Thromboembolic Disorders and Antithrombotic Therapies, University of Insubria, Varese, Italy; 3 U.O.C. Medicina Generale, Ospedale Sant’Anna - ASST Lariana, via Ravona 20, 22042 San Fermo della Battaglia, Como Italy; 4 Department of Medicine and Surgery, University of Insubria, Como, Italy

## Correction to: Internal and Emergency Medicine 10.1007/s11739-019-02177-2

The article Reversal agents for oral anticoagulant-associated major or life-threatening bleeding, written by Marco Moia, Alessandro Squizzato was originally published electronically on the publisher’s internet portal (currently SpringerLink) on 24 August 2019 without open access.

With the author(s)’ decision to opt for Open Choice the copyright of the article changed on 30 November 2019 to © The Author(s) 2019 and the article is forthwith distributed under the terms of the Creative Commons Attribution 4.0 International License (http://creativecommons.org/licenses/by/4.0/), which permits use, duplication, adaptation, distribution and reproduction in any medium or format, as long as you give appropriate credit to the original author(s) and the source, provide a link to the Creative Commons license and indicate if changes were made.

**Open Access** This article is distributed under the terms of the Creative Commons Attribution 4.0 International License (http://creativecommons.org/licenses/by/4.0/), which permits unrestricted use, distribution, and reproduction in any medium, provided you give appropriate credit to the original author(s) and the source, provide a link to the Creative Commons license, and indicate if changes were made.

The original article was corrected.

